# The Impact of SARS-CoV-2 Infection on Youth Mental Health: A Narrative Review

**DOI:** 10.3390/biomedicines10040772

**Published:** 2022-03-25

**Authors:** Claudio Brasso, Silvio Bellino, Cecilia Blua, Paola Bozzatello, Paola Rocca

**Affiliations:** Department of Neuroscience “Rita Levi Montalcini”, University of Turin, 10126 Turin, Italy; silvio.bellino@unito.it (S.B.); cecilia.blua@unito.it (C.B.); paola.bozzatello@unito.it (P.B.); paola.rocca@unito.it (P.R.)

**Keywords:** SARS-CoV-2, COVID-19 pandemic, syndemic, mental health, youth, depression, anxiety, suicidal thoughts, social media, telemedicine

## Abstract

Background: COVID-19 pandemic has affected the physical health, psychological wellbeing, and mental health of the whole population. Young people are among those most at risk of developing mental health symptoms or disorders related to the pandemic. Purpose: the present narrative review is aimed at providing an updated overview of the current literature concerning the psychological impact of the SARS-CoV-2 infection but also of the COVID-19 outbreak, environmental restriction, and social distancing on mental health outcomes among the youth population aged between 15 and 25 years. Methods: in December 2021, an electronic search on this topic was performed on PubMed. Relevant publications from January 2020 until December 2021 were included. Findings: 53 cross-sectional studies, 26 longitudinal studies, 4 ecological studies, 1 qualitative study, and 1 systematic review were included. We found many methodological limitations in the studies included, especially poor choice of study samples and short follow-ups. Little literature was in support of a strong relationship between SARS-CoV-2 infection and consequences on youth mental health. On the contrary, many studies showed how extraordinary measures to limit the spread of the virus have impacted young people in terms of onset of new mental disorders and symptoms, suicidality, and access to emergency psychiatric services. Depressive and anxiety symptoms and disorders show the greatest increase in incidence, especially in girls and young women. Conclusions: it seems important to pay attention to the mental health of young people in relation to the consequences of the COVID-19 pandemic. However, studies with more robust methodologies and longer follow-ups are needed to establish precise indications for targeted interventions in this context.

## 1. Introduction

In December 2019, a cluster of four cases of pneumonia of unknown etiology in Wuhan, China, were reported to the World Health Organization (WHO) [1]. Since then, coronavirus disease 2019 (COVID-19), caused by severe acute respiratory syndrome coronavirus 2 (SARS-CoV-2), has spread rapidly across the world. SARS-CoV-2, according to available data, can infect various organs and rapidly proliferate, triggering inflammatory reactions in the human body [2]. The SARS-CoV-2 infection can be completely asymptomatic or, conversely, can cause COVID-19, with a clinical spectrum ranging from mild upper respiratory tract symptoms to severe pneumonia and acute respiratory distress syndrome (ARDS) or acute respiratory failure [3,4,5,6]. Besides respiratory symptoms, neurologic and neuropsychiatric complications have been increasingly reported [7,8]. Furthermore, persistent and prolonged symptoms, similarly to previous coronavirus outbreaks [9], have been observed in patients recovered from the acute phase [10,11], and constitute what is called post-acute COVID-19 syndrome. Reports at three, four, and six months following discharge suggested persisting symptoms as lung dysfunctions and physical problems, but also psychological disturbances and cognitive impairments [11,12] Moreover, histopathologic examination of brains from deceased COVID-19 patients indicate the potential of SARS-CoV-2 to infiltrate the central nervous system (CNS) [13]. Reported neuropsychiatric manifestations include milder symptoms like dizziness and anosmia [14], in rare cases severe manifestations such as acute demyelinating encephalopathy [15], meningitis [16], strokes [17,18], but also cognitive and attention deficits (“brain fog”), new onset anxiety, depression, psychosis, and suicidal behavior [19,20,21,22,23]. 

In addition to the biological effects of the SARS-CoV-2 infection on the CNS, after the outbreak of the pandemic state [24], every country in the world has restricted freedom of movement and limited non-emergency health care to focus resources on COVID-19 care provision [25]. The pandemic has produced a devastating impact on the global economy and the health of communities across the world [26,27] and has unleashed and amplified many simultaneous personal, social, medical, political, and economic crises. To describe how COVID-19 clusters with these pre-existing conditions and interacts with them, the concept of syndemic has been proposed [28]. Syndemic is not merely a comorbidity, but it is characterized by “biological and social interactions between conditions and states, interactions that increase a person’s susceptibility to harm or worsen their health outcomes” [29]. From this perspective, the impact of the COVID-19 pandemic should be considered in terms not only of significant morbidity and mortality directly caused by the SARS-CoV-2 infection but also of its indirect effects on health, particularly on mental health. Several studies reported that measures to counter the COVID-19 pandemic and consequent quarantine’s periods affected mental health in both clinical and general populations [30,31,32,33,34,35,36] and that good stress response and a positive appraisal are the strongest resilience factors during the pandemic [37]. Moreover, the social distance imposed to limit the spread of the virus and the consequent reduction in social interactions impacted social behavior as well modulating neural activation [38,39].

In particular, fears connected with a new disease and limitations of social interactions especially influenced the lives of adolescents and young adults who experienced a period of increased vulnerability to mental health problems. Adolescence is a particular window of time in the psychological development of an individual in which somatic changes, intrapsychic events, and psychosocial dynamics are inextricably intertwined and interdependent in laying the foundations for the individual’s psychological development. Furthermore, several mental illnesses, including primary psychosis, bipolar disorder, depression, anxiety, eating disorders, personality disorders, and addictive disorders, first appear before 24 years of age [40]. This makes adolescence both a time of extreme vulnerability and opportunities for action. In this regard, a disruptive event, such as a pandemic, with the resulting measures to counter it may interfere with this complex life period by limiting possibilities and at the same time by introducing a burden of suffering on multiple levels: personal, social, and environmental. Despite acute COVID-19 illness being milder in the young population, it should not be assumed that those at low risk of life-threatening acute infections [41,42] do not suffer neurological and psychiatric consequences of SARS-CoV-2 infection. For this reason, it is mainly important to analyze and understand the effects of the COVID-19 pandemic on the adolescent/young adult population.

In the present work, we will focus first on the impact of the viral infection on the CNS and its possible consequences in terms of mental health of young people, then on the indirect syndemic effects of the virus widespread. The mental health outcomes in young people will be grouped in four sections: the first about the biological sequelae of the SARS-CoV-2 infection on the CNS, the second concerning the onset of new mental disorders and symptoms, the third relating to suicidality and access to emergency psychiatric services, and the fourth regarding the role of technology and social media. 

The aim of the present narrative review is to provide an updated overview of the current literature concerning the psychological impact of the SARS-CoV-2 infection but also of the COVID-19 outbreak, environmental restriction, social distancing, and extraordinary measures to restrain the spread of infection, on mental health outcomes among the youth population aged between 15 and 25 years. 

The objective of this work is to provide a concise, but complete picture of the direct and indirect impact of the SARS-CoV-2 virus on the mental health of young people to provide clinicians, not necessarily mental health experts, the tools for the early detection of signs of distress in a fragile stratum of the population, that needs to be protected and supported during this pandemic period to achieve a healthy psychological development towards adulthood.

We expect to find mainly results on specific aspects related to the impact of the first wave of the pandemic. We believe that few studies focused on wider issues or described longer follow-ups. These expectations relate to two factors: on the one hand, the short time elapsed since the outbreak of the pandemic at the time of our bibliographical research, and, on the other, the pressure for publication in relation to the emergency.

## 2. Materials and Methods

In December 2021, an electronic search was performed on PubMed about the effects of the COVID-19 pandemic on the adolescent/young adult population using the following search string ((“Mental Health” (Mesh)) OR (“Mental Disorders/diagnosis” (Majr)) OR “Mental Disorders/epidemiology” (Majr) OR “Mental Disorders/statistics and numerical data” (Majr) OR “Suicide, Attempted/statistics and numerical data” (Majr)) OR ((“post-acute COVID-19 syndrome” (Supplementary Concept)) AND “Psychiatry and Psychology Category” (Mesh)) AND ((“COVID-19” (Mesh) OR “SARS-CoV-2” (Mesh)) AND (“Adolescent” (Mesh) OR “Young Adult” (Mesh))).

We included the following types of publication: observational studies (cross-sectional studies, cohort studies, and ecological study), longitudinal and prospective studies, surveillance studies, reviews, and meta-analyses from 1 January 2020 until 31 December 2021. Overlapping studies were excluded. The review considered only articles written in English.

## 3. Results

The search described in the methods provided 1118 records. Eligibility status for articles was determined according to the following way: (1) all studies were screened on the basis of the title and abstract; and (2) papers that passed the initial screening were reviewed on the basis of a careful examination of the full manuscript content. This review included 85 records, 84 observational studies (53 cross-sectional studies and 26 longitudinal studies, 4 ecological studies, and 1 qualitative study), and 1 systematic review.

## 4. Discussion

### 4.1. Biological Sequelae of the SARS-CoV-2 Infection on the CNS

The pathogenesis of psychiatric symptoms and disorders that arise during and after the SARS-CoV-2 infection concerns mainly indirect effects of the virus on the CNS and is currently being debated. In this section we will focus on: (1) the putative biological mechanism that could cause psychopathological manifestations in the youths, and (2) the main psychiatric symptoms observed in this population during and after the infection. 

#### 4.1.1. Putative Biological Mechanism of Psychopathological Manifestations of SARS-CoV-2

**Figure 1:**
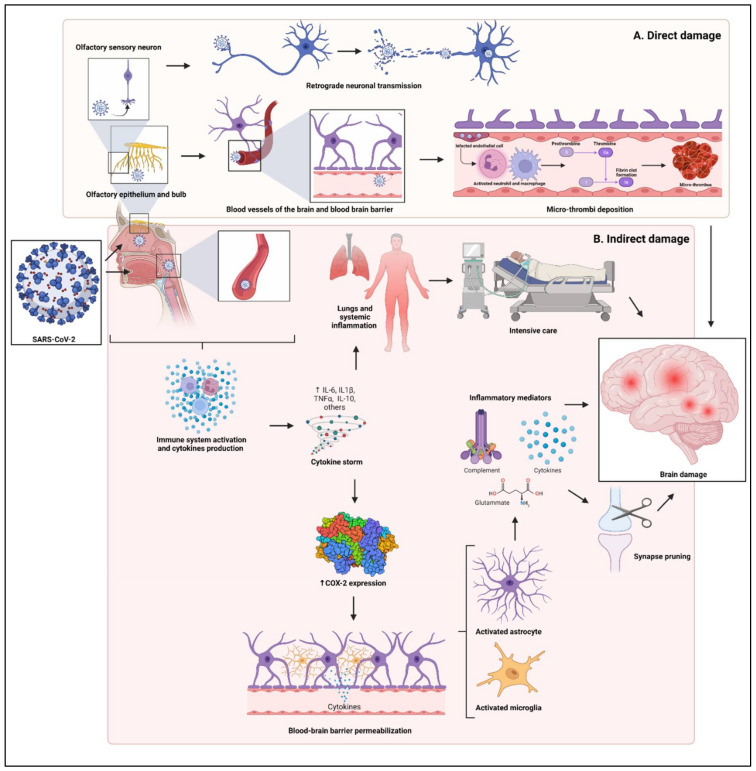
Possible mechanisms of direct and indirect SARS-CoV-2 infection damage to the brain. (**A**) Direct damage: SARS-CoV-2 infects the olfactory nerve and then the olfactory bulb. Via retrograde neuronal transmission, it reaches other CNS regions [44,45,46] (upper part of panel (**A**)). It infects endothelial cells of the brain vascular system and activate neutrophils, macrophages, thrombin production, and complement pathways, promoting micro-thrombi deposition [48] (lower part of panel (**A**). (**B**) Indirect damage: SARS-CoV-2 spreads to the whole organism and causes a massive activation of the immune system. This results in a cytokines storm and in a marked systemic inflammatory response [53,54]. An overactive immune response can cause lungs damage and the need for intensive care. Aggressive life-saving treatments in intensive-care units may contribute to the brain damage [11] (upper part of panel (**B**)). Cytokines induces expression of cyclooxygenase 2 (COX-2). The activity of COX-2 increases blood-brain barrier permeability. This facilitates the entry of cytokines within the CNS, which causes the activation of astrocytes and microglia [55,56,57]. These activated cells produce mediators of neuro-inflammation (e.g., glutamate, complement proteins, and interleukins), that induce structural changes, in particular, the pruning of the synapses [57,58] (lower part of panel (**B**). Both direct and indirect brain damages can converge in a neuronal loss that might represent the biological underpinning of the neuro-psychiatric symptoms described in SARS-CoV-2 infection [11,52] (not shown). SARS: severe acute respiratory syndrome; CoV: coronavirus; IL: interleukin; and TNF: tumor necrosis factor. Created with BioRender.com.

Some authors suggest that SARS-CoV-2 produces direct damages to the CNS through both neuronal and endothelial cells infection [43]. The virus can infect peripheral neurons and exploit axonal transport pathways [44,45,46]. It is mostly transmitted through the nasal mucosa where it reaches the olfactory nerve and then the olfactory bulb that provides access to other CNS regions via retrograde neuronal transmission [47,48]. Although there is no consensus on the consequences of this infection, it is possible that it causes local hypoxia, lowering the threshold for tissue damage in the context of a pre-existing oxygen-deprived state [49]. In addition, the SARS-CoV-2 may infect endothelial cells of the brain vascular system and activate neutrophils, macrophages, thrombin production, and complement pathways, promoting micro-thrombi deposition [48] (Figure 1A). This mechanism is confirmed by the fact that several recent tissue-based studies in fatal COVID-19 cases have reported micro–hypoxic/ischemic injuries of the CNS [13,15,50,51,52]. 

Together with the direct biological damage to the CNS, an important role can be played by the immune response associated with the coronavirus disease 2019 (COVID-19). In fact, dysregulated and overactive immune response, which is commonly observed in patients with COVID-19 [59,60], may indirectly affect the CNS through the inflammatory immune response [17,53,54,61,62,63]. A significant cytokine storm has been described in patients with severe COVID-19, with elevated serum levels of pro-inflammatory cytokines such as interleukin (IL) 1, IL-6, IL-10, and tumor necrosis factor (TNF)-α. These molecules increase the blood-brain barrier permeability via the cyclooxygenase-2 upregulation, allowing pro-inflammatory cytokines themselves to enter the CNS [55,56] with the activation of astrocytes and microglia and the release of inflammatory mediators (glutamate, complement proteins, interleukins, quinolinic acid, and acute-phase proteins). This cascade leads to neuroinflammation and contributes to structural changes, in particular, the pruning of the synapses [57,58] (Figure 1B). Neuroinflammation was observed in severe psychiatric disorders [64], such as major depressive disorder [65,66,67,68,69,70,71,72], schizophrenia [73,74,75,76], and ADHD [77]. 

In conclusion, microvascular thrombosis and immuno-mediated damages can converge in a neuronal loss that might represent the biological underpinning of the neuropsychiatric symptoms described in SARS-CoV-2 infection [11,52]. However, especially for depressive, post-traumatic, and neurocognitive symptoms, it is extremely difficult to discriminate between the direct and indirect biological effects of the virus, iatrogenic effects related to aggressive life-saving treatments in intensive-care units, and psychological trauma related to the infection itself [11].

#### 4.1.2. Neuropsychiatric Symptoms in Youth during and after the SARS-CoV-2 Infection

Although young people are generally less severely affected by acute COVID-19 than elder adults, it should not be assumed that subjects at low risk of life-threatening acute infections [41,42] do not suffer from neuropsychiatric sequelae of the SARS-CoV-2 infection. For this reason, the evaluation of the effects of COVID-19 on youths’ mental health (population aged between 15 and 25 years) is a relevant issue that requires further discussion. To date, studies that analyzed this specific subpopulation are scarce, probably due to less need for hospitalization of young patients with COVID-19 and less opportunity to monitor them into follow-up studies. 

Notwithstanding the limited number of studies about this topic, in this subsection, we will summarize the main findings on neurocognitive impairment, post-traumatic, depressive (especially fatigue), and anxiety symptoms in young people who were affected by the COVID-19 disease (Table 1). 

One online study from the Imperial College in London [80] analysed data from 84,285 individuals who completed a questionnaire to determine whether those who had recovered from COVID-19 reported objective cognitive deficits. The authors reported that, regardless of the age of the participants, the subgroup between the ages of 20 and 70 that was hospitalized with ventilator showed cognitive impairment equivalent to a 10-year decline in global performance. According to Hampshire et al., young adults as early as in their 20s showed large cognitive impairments in multiple domains, particularly semantic problem solving, visual attention, and executive functions. 

Results from a study performed by Blomberg et al. supported similar conclusions [23]: they conducted a long-term follow-up in a prospective cohort study of 312 patients (247 home-isolated and 65 hospitalized). At 6 months, 52% of home-isolated young adults, aged 16–30 years, reported fatigue (21%), impaired concentration (13%), and memory problems (11%). 

Another study conducted on 126 COVID-19 survivors aged from 11 to 72 (mean = 45.7, SD = 14.0) evaluated depression, anxiety, and PTSD symptoms [78]. Authors did not find significant differences between the older COVID-19 survivors and the younger survivors for depression symptoms, while younger survivors reported more severe stress response symptoms and anxiety symptoms than older subjects, indicating that younger participants have more emotional reactivity to infection.

An interesting cohort study carried out by Roge et al. at the Children’s Clinical University Hospital in Latvia compared the long-term sequelae of COVID-19 patients with other non-SARS-CoV-2-community-acquired infections [81]. A total of 236 pediatric COVID-19 patients and a comparison group of 142 patients were enrolled in the study. Psychiatric complaints that were commonly observed among COVID-19 patients included persistent fatigue (25.2%), irritability (24.3%), and mood changes (23.3%). In addition, 105 (44.5%) COVID-19 patients had persistent symptoms after the 12-week cut-off point, with irritability (27.6%), mood changes (26.7%), and fatigue (19.2%) being the most commonly reported ones. Interestingly, the prevalence of persistent fatigue and cognitive symptoms significantly increased according to the study’s age groups: the highest rates were found among teenagers (14.7%, 1–4-year-olds versus 37.0%, 15–18-year-olds). Among them, the most reported persistent symptoms were cognitive disturbances, including difficulty in concentrating (27.8%) and an inability to focus their attention (24.1%). Moreover, the comparison between the SARS-CoV-2 infection with any other non-SARS-CoV-2 identified that symptoms persistence is more apparent with COVID-19 disease. 

A different perspective was presented by Blankenburg and colleagues [79], that surveyed 1560 students with a median age of 15 years: 1365 (88%) were seronegative while 188 (12%) were seropositive for SARS-CoV-2 infection. All participants completed a post-acute COVID-19 syndrome survey regarding the occurrence and frequency of concentration difficulties, memory loss, listlessness, headache, abdominal pain, myalgia/arthralgia, fatigue, insomnia, and mood alterations (sadness, anger, happiness, and tenseness). Authors found a high rate of neurocognitive, pain, and mood symptoms in the group of adolescents. Symptoms most frequently reported were insomnia, pain, fatigue, and concentration difficulties, but an equal prevalence of neurocognitive, pain and mood symptoms were registered in seronegative and seropositive adolescents. These findings did not negate the existence of post-acute COVID-19 symptoms in general or in the youth but emphasized the role of pandemic-associated symptoms (e.g., social distancing) in the adolescents’ well-being and mental health. 

In summary, the evidence about post-acute COVID-19 cognitive and psychiatric symptoms in youth is limited. Further investigations are needed to distinguish symptoms directly related to the SARS-CoV-2 infection from pandemic-associated complaints, especially in a critical age group such as adolescents and young adults. However, the possible medium- and long-term effects of SARS-CoV-2 infection on the CNS and the mental health of young people should be taken into account for the assessment of future health policies.

### 4.2. The Role of COVID-19 Pandemic in the Mental Health of Young People: Onset of New Mental Disorders and Symptoms 

Many original contributions have been concerned with the development of psychological and/or behavioral signs and symptoms following the outbreak of the COVID-19 pandemic while few epidemiologic studies have focused on the variation in the incidence or prevalence of mental disorders in the population of young people. In this section, we will summarize and discuss these studies dividing them into two groups following these two main topics (i.e., incidence of new mental symptoms or new mental disorders). 

#### 4.2.1. Onset of New Symptoms or Signs Affecting the Mental Health of Non-Clinical Populations of Young People 

According to the World Health Organization, mental health is defined as “a state of well-being in which every individual realizes his or her own potential, can cope with the normal stresses of life, can work productively and fruitfully, and can contribute to her or his community.” [82,83,84,85,86,87,88,89,90,91,92,93,94,95,96,97,98,99,100] (WHO, 2018). So, it is intuitive to discern how the pandemic has had an impactful role on the mental health and emotional well-being of millions, regardless of nationalities. Since early 2020, due to the ongoing COVID-19 pandemic, citizens from around the world have been experiencing stressful events such as unemployment resulting in loss of income, sickness, social isolation, death of a family member due to the virus, the uncertainty of what the future holds, helplessness, and lack of individual control over the situation. These difficulties are particularly marked among young individuals. The lack of contact with peers, disruption in education, and, for many, the loss of emotional and financial security, impacted daily lives. This has resulted in youth facing an increased risk of suffering from mental health problems. 

The studies we reviewed that addressed the impact of COVID-19 syndemic on mental health of the general populations of adolescents and young adults showed that, during the COVID-19 outbreak, youth experienced depressive symptoms [82,83,84,85,86,87,88,89,90,91,92,93,94,95,96,97,98,99,100]; anxiety symptoms [82,83,84,85,86,87,88,89,90,91,93,94,95,96,97,98,99,100,101,102,103,104,105,106,107,108,109,110]; distress [84,85,86,91,93,96,98]; post-traumatic stress symptoms [94,111]; obsessive compulsive symptoms [89,105]; eating disorder symptoms [112,113,114,115,116,117,118,119]; and changes in sleep pattern [94,97,99,109,120], and substance abuse [100,121,122]. 

In addition, two studies and two case reports examined incident cases of primary psychosis during the COVID-19 pandemic [123,124]. 

##### Depressive Symptoms 

Available evidence highlighted that young people experienced depression symptomatology during the COVID-19 outbreak. As regards the targeted sample, we observed that most studies were performed on college or university students [83,84,85,86,87,93,96,97,98,99]. The other samples consisted of adolescent students [82,88,89,90,91,92] or general population [94,95,100].

The prevalence of depressive symptoms in young people across the studies that reported such information ranged from 21.1% [93] to 82.4% [83]. Both studies used the 9-item Patient Health Questionnaire (PHQ-9) to measure students’ depressive symptoms, a questionnaire also used by most of the clinical studies included in this review [83,87,88,90,93,94,95,96,98,99]. The differences in prevalence might be due to the different cut-off points: for the first study a total score of 7 was used as a cut-off point to screen clinical depressive symptoms, the second one chose a total score of 5. In this way, the cut-off score of the latter study has been found to be more inclusive. Many other studies used the Depression Anxiety Stress Scale (DASS-21) [84,85,86,91]; reporting a prevalence of depression symptoms that ranged from 28.8% [91] to 46.92% [84]. 

In three studies assessing students’ samples, depressive symptoms were related to concerns about academic performance being impacted by the pandemic [83,87,97]. One study conducted in the USA [97] showed that the biggest perceived challenge was the transition to online classes (38% of the sample). 23% of participants were worried about progress in research and class projects because of the lack of physical interactions with other students. Some participants (14%) mentioned the uncertainty about their grades under the online learning environment to be a major stressor. 8% of the sample pointed out lack of motivation to learn and tendency to procrastinate. 

It’s interesting to note that in many cases higher levels of depressive symptoms were positively associated with excessive exposure to COVID-19 news in social media [84,92,93,98] and female gender [88,90,96,98,99]. 

On the contrary, physical exercise [84,86], working [96], and perceived social support, both from families and friends [90,95,98], have been associated with a lower risk of developing depressive symptoms in the young population. 

##### Anxiety Symptoms 

Several studies established a significant association between the COVID-19 pandemic and rates of anxiety among young people. The majority of studies we found concerned college or university students [83,84,85,86,87,93,96,97,98,101,102,103,104,105,106,108,110,125]. The other samples consisted of adolescent students (mostly high school students) [82,88,89,90,91,107] and the general population stratified for age [94,95,100]. The prevalence of anxiety symptoms in young people ranged from 11.1% [93] to 87.7% [83]. Both studies used the 7-item Generalized Anxiety Disorder Scale (GAD-7) to measure anxiety symptoms, a questionnaire widely used in clinical studies on this topic [83,87,88,90,93,94,95,96,98,101,103,104,108,125]. As we have stressed before, the differences in prevalence might depend on the different cut-off points used: the study conducted by Ma et al. [93] used a cut-off score of 7 to screen clinical anxiety symptoms, while the one led by Islam et al. [83] used a cut-off score of 5, turning out to be more inclusive. The longitudinal study performed by Parola et al. [82], showed an increase in the levels of anxiety while the lockdown measures were in place. 

Among risk factors for developing anxiety symptoms, another longitudinal study [106] reported that individual responses to the quarantine are influenced by the “proneness to worry” trait. Young people who were “high worriers” were more anxious during the pandemic outbreak.

Other investigations were focused on the living condition of the young people in relation to the levels of anxiety during the lock-down [86,103]. Authors found that high anxiety levels were associated with living in an urban area, having no direct outside access through a garden, a terrace, or a balcony, living in noisy environments, having difficulties in isolating, and tensions or conflicts with family or occupants of the dwelling [94].

Anxiety was also associated with concerns about financial situations being impacted by COVID-19 [83,84,97,98,101,110,125] and social media exposure [84,93,98,100]. Among the students, challenges of remote learning, the delay of final examinations, uncertainty related to exam dates, and concern about their academic performance were found to be risk factors for anxiety symptoms [83,97,101,102,103,104,105,110,125]. One study also has suggested that healthcare and medical students had a lower risk of developing anxiety compared with students in other fields of study [110].

As for depressive symptoms, the female gender was associated with a higher level of anxiety during the COVID-19 pandemic compared to males [87,90,91,102,103,110]. Different studies also have underlined that high levels of anxiety were significantly different according to age as younger individuals experienced more anxiety than older ones [85,87,94,110].

By contrast, living in rural areas [86] and proactive healthy behaviors, such as physical exercise or meditation have been associated with lower rates of anxiety among youth [84,86,90,97,98,99].

##### Distress

Several studies reported that young subjects experienced psychological distress because of the COVID-19 outbreak and lockdown conditions [84,85,86,93,96,97,125]. The distress prevalence in young people across the studies ranged between 22% [85] and 69.3% [84]. Differences in the distress prevalence might be due to the difference in evaluation instruments. In particular, the prevalence of distress ranges between 22% [85] and 28.5% [84], when assessed with the Depression Anxiety Stress Scale (DASS-21), and between 34.9% [93] and 69.3% [84], when measured with the Impact of Event Scale (IES). 

In addition, one study showed that younger people experienced more stress than older people (>35 years) [85], while the study conducted by Khan et al. [84] reported that students aged above 25 had higher distress levels than younger students. Furthermore, female gender [91,96], living in an urban area, living with families [86], sedentary lifestyle, financial uncertainty [84,98], and excessive exposure to social and mass media [84,93] were associated with higher levels of psychological distress. 

##### Post-Traumatic Stress Disorder Symptoms

Two studies reported symptoms of post-traumatic stress disorder (PTSD). The first one was conducted in China by Liang and collaborators [111]. They enrolled a total of 584 youth aged between 14 and 35 years who were tested with the PTSD Checklist—Civilian Version (PCL-C). About 14% of the youth presented PTSD symptoms. Men were significantly more prone to develop PTSD in comparison with woman. Participants with lower education were more likely to show PTSD symptoms and psychological distress. In addition, divorced/widowed participants had significantly higher PTSD scores than single and married/cohabitating participants. Businesspeople have shown significantly higher PTSD symptoms scores as compared to people working in other fields. The last findings might be explained by the fact that, during the occurrence of COVID-19, the business people who needed to trade with others were worried about being infected by the SARS-CoV-2 and developed more PTSD symptoms.

The second study was performed by Murata and Colleagues [94]. They recruited 4909 participants through an online survey from 27 April to 13 July 2020. They tested participants with Primary Care PTSD Screen for DSM-5 (PC-PTSD-5) and they reported that adolescents were significantly more likely to report moderate to severe symptoms of PTSD (45% versus 33%) than adults. The most common trauma reported was sickness or death of a loved one (48%); sexual, physical, or domestic abuse (7%); accidents, disasters, violent crimes, and mass shootings (21%), suicide of a loved one (6.8%); abandonment, emotional abuse, divorce or breakup (4.9%); and other traumas (10.7%), such as legal and financial problems. 

In the subsample of the adolescents, female sex, loneliness, lifetime history of suicidal ideation, and health worries about COVID-19 predicted PTSD symptoms. Moreover, female adolescents with higher household income in the past 12 months showed lower PTSD symptoms.

On the other hand, in the subsample of adults, factors associated with higher levels of PTSD symptoms were male sex, loneliness, being older age with chronic medical diseases, belonging to an ethnic minority, having a lower subjective social status, or sleep problems. Moreover, when including health care workers’ exposure variables, worries about getting infected due to work exposure predicted PTSD symptoms. 

##### Obsessive-Compulsive Symptoms 

Two studies examined the relationship between COVID-19 and obsessive-compulsive symptoms. The first one was conducted by Seçer and colleagues [89] in a Turkish sample of 598 adolescents. They showed that fear of COVID-19 has a positive and significant effect on OCD symptoms (reduction of symptoms) which is mediated by emotional reactivity, experiential avoidance, and depression–anxiety.

The second study was performed by Jiang et al. [105] testing 511 students from a university in China assessed using the COVID-19 General Information Questionnaire and the Symptom Checklist 90 (SCL-90) questionnaire. The scores assessing obsessive-compulsive disorder were significantly increased compared with the norm. However, no differences in the scores for depression, hostility, and psychoticism were noted (*p >* 0.05).

##### Eating Disorder Symptoms 

Many studies collected evidence regarding the impact of the SARS-CoV-2 pandemic on eating disorder (ED) symptoms. We selected and examined eight studies sampling a non-clinical population of young people [112,113,114,115,116,117,118,119]. Each of these studies found a sizeable percentage of participants reporting specific ED symptoms due to the pandemic, including dietary restriction [115,118], increased food consumption [118] and binge eating [115,116,118], eating to cope [112,118], purging [115], decrease in physical exercise [114,117,119] or excessive exercising [115], concerns with eating, shape, and weight [113,114,115,118] and weight change [115]. The reviewed studies included university and college students [114,115,117], adults from the community [112,116,118], and special populations, such as health club users [119] and former athletes [113]. One study has underlined that the female gender was associated with a higher level of eating disorder symptoms compared to the male one [113]. 

Across ED-related aberrant behaviors, physical exercise is worth of a specific mention. Indeed, pandemic restrictions reduced the possibility to do physical activity: this promoted a high variability in the amount of physical exercise performed by the people concerned about their weight. Keel and colleagues [114] indicated that American university students they tested reported a decrease in physical activity along with increased concerns about weight, shape, and eating. This is in line with another study conducted among university students [117] that reported a significant decrease in exercise associated with COVID-19. In contrast, the study conducted by Kohs and Collaborators [115] among German university students, highlighted that 28.0% of the sample reported excessive exercising minimum once per week, but 26.2% of the surveyed sample reported weight gain. A study conducted among health club users [119] reveals that exercise addiction scores were significantly lower post-lockdown but leisure-time exercise was significantly increased. As regards risk factors, depressive and anxiety symptoms [118], financial difficulties [118], and intolerance of uncertainty [117] were significantly associated with ED symptoms. 

Special attention should be paid to the study performed by Puhl and colleagues [116] who examined longitudinal associations between pre-pandemic experiences of weight stigma and eating behaviors, psychological distress, and physical activity during the COVID-19 pandemic in a sample of young adults. They reported that young adults who have experienced weight stigma may have increased vulnerability to adverse health behaviors, such as maladaptive eating during the pandemic. 

##### Sleep Disturbances

The COVID-19 outbreak, lockdown restrictions, and quarantine measures also influenced changes in sleep habits. The increase of sleep problems reported by the studies ranged from 11% [109] to 86% [99] among studies. The decrease in sleep quality was stronger for people who were younger; with a higher level of education, non-married, affected by any chronic medical disease [99]. Moreover, a significant association was found between worse sleep quality and depression and higher levels of anxiety [120].

##### Changes in Alcohol and Substance Use 

We collected three studies that provide information on how adolescents’ substance use has changed since the COVID-19 outbreak.

An online survey conducted in Canada among 1054 adolescents (Mean age = 16.7 years) reported the frequency of alcohol and cannabis use increased but, for most substances, the percentage of users decreased. The greatest percentage of adolescents was engaging in solitary substance use (49.3%), while 31.6% were still using substances with peers via technology, and 23.6% of the sample face to face despite the rules of social distancing [121]. 

Czeisler and collaborators [122] tested 5412 adults through web-based surveys during 24–30 June 2020. They reported that 13.3% of subjects, in particular, those with an age range between 18 and 24 years, increased substance use to cope with stress or emotions related to COVID-19. An increase in substance use was also associated with being employed, being black, and being an unpaid caregiver for adults. 

Different findings were reported by Glowacz and colleagues [100], that tested 2871 adults with an online questionnaire during the lockdown and reported that young adults presented lower levels of alcohol use than older participants. According to the authors, this result can be explained by the fact that alcohol use among the young mainly takes place in social contexts, whereas older people increase their alcohol use to cope with the lack of contact.

#### 4.2.2. Variation of the Incidence or Prevalence of Mental Disorders in the Populations of Young People after the Onset of the COVID-19 Pandemic

Two studies [123,124] examined the variation of incidence of psychosis during COVID-19 pandemic among young population. Esposito [124] compared 62 patients hospitalized for first-episode psychosis (FEP) between 8 March to 8 July 2020, versus subjects first hospitalized in the same period in 2019. He reported a 29.6% increase during the identified timeframe compared to the previous year. Patients hospitalized for a FEP in 2020 were found to be significantly older (mean age: 43.5 versus 34.0), and to report significantly lower substance abuse behaviour than patients hospitalized the previous year (17.1% versus 59.3%,). Wu and colleagues [123] surveyed 1825 adolescents before (20 October 2019) COVID-19 and after (18 May 2020) the lockdown in China. Psychotic-like experiences (PLEs), anxiety, and depression were measured with paranoia, anxiety, and depression subscales of the Mental Health Inventory of Middle school students (MMHI-60). They reported a significant increase in adolescent PLEs after the lockdown. They also identified four PLEs trajectories based on the report of PLEs at two time points: 60.4% with no PLEs, 9.3% remitted PLEs, 16.7% new PLEs, and 13.6% persistent PLEs. Notably, the group with new-onset PLEs had the greatest exacerbation in anxiety/depression symptoms.

A recent systematic review (COVID-19 Mental Disorders Collaborators 2021) [126] that has included 46 studies on major depressive disorder and 27 on anxiety disorders from 204 countries reported that daily SARS-CoV-2 infection rates and reductions in human mobility were associated with the increased prevalence of both groups of disorders. Moreover, females and younger age groups (15–30 years old) showed a higher prevalence of major depressive disorder and anxiety disorders after the COVID-19 outbreak. 

As regards eating disorders (ED), a study by Taquet and Collaborators [127] (2021) performed on the electronic health records of 5.2 million people aged under 30 years, mostly in the USA, has shown that the diagnostic incidence of EDs was 15.3% higher in 2020 compared with previous years. They have also reported that the relative risk increased steadily from March 2020 onwards exceeding 1.5 by the end of the year. This increase occurred solely in females, especially in teenagers.

The results of this section are summarized in the following table (Table 2).

### 4.3. Suicide-Related Issues in the Youth during the COVID-19 Pandemic

Suicide represents the second-leading cause of death among 15- to 29-year-olds worldwide [128]. Suicidal behaviors include ideation (thoughts and plans of ending one’s life) and attempts (engagement in potentially self-injurious behavior that does not result in death) [129]. Suicide-related issues, such as suicidal ideation, constitute a significant risk factor for suicidal attempts and death [130,131]. Socioeconomic adversity, adverse childhood events, bullying victimization, substance abuse, and psychological problems are identifiable predictors of the development of adolescent suicidal ideation [124,130,131,132].

In recent world history, major infectious outbreaks were linked to severe mental health outcomes, including suicide. For instance, a study conducted by Wasserman in 1992 showed that suicide rates increased in the USA during the 1918–19 influenza pandemic [133]. Deaths by suicide increased also in Hong Kong during the 2003 Severe Acute Respiratory Syndrome (SARS) epidemic [134]. Nowadays, the global pandemic of COVID-19 has provided significant challenges to people’s quality of life, placing stress on financial, occupational, academic, social, physical, and emotional well-being. So, the risk of suicidal behaviors among the vulnerable individuals (i.e., history of presence of psychiatric symptoms, poverty, and poor living conditions) could be increased [135]. 

In this subsection, we will summarize the literature on suicide-related issues in young people in relation to the COVID-19 pandemic and the impact of this phenomenon on psychiatric emergency services (Table 3).

#### 4.3.1. Suicide Ideation 

As regards the change of suicidal ideation status among young people, Hill and colleagues [145] examined rates of suicide ideation and attempts reported during routine suicide risk screening in a pediatric emergency department in Texas. They compared rates from January–July 2020 with corresponding rates from 2019 examining a total of 9092 screens. Participants had a mean age of 14.52 years (SD = 2.22; with 88.8% aged 11–17 years). Comparison of screens indicated a significantly higher rate of suicide ideation in March and July 2020 and higher rates of suicide attempts in February, March, April, and July 2020 than the same months in 2019. They noticed that rates of suicide ideation and attempts were higher during some months of 2020, as compared with 2019, but were not universally higher across this period: months with statistically significantly higher rates of suicide-related behaviors matched times when COVID-related stressors were intensified. We have to consider that the number of emergency department visits was substantially decreased during the COVID-19 pandemic, so a direct comparison of rates across years should be made with caution. 

These results are in line with another study conducted by Czeisler et al. [122] in the USA to investigate the trend of suicidal ideation during the pandemic period. Authors stated that, in June 2020, 25% of the 5470 young adults aged 18–24 years reported experiencing suicidal ideation related to the pandemic in the previous 30 days, twice more than in 2018 (10.7% versus 4.3%). This net difference could depend in part on the study design: the retrospective nature and the self-assessment of suicidal ideation might underestimate suicidal thoughts prior to the pandemic outbreak and/or overestimate them during the pandemic.

In Canada, 809 adolescents [151] aged 12–18 years were recruited via social media advertisements between June and July 2020 and were tested for the presence of suicide ideation. Results showed that 44% of adolescents experienced suicide ideation since the pandemic began, while 32% reported engaging in deliberate self-harm. Suicidal ideation and deliberate self-harm were more common among youth who identified as transgender, gender fluid, or non-binary; who did not reside with both parents; and who reported frequent cannabis use or psychiatric concerns. 

In contrast to these findings, the study performed by Gratz and collaborators [144] examining rates of lifetime and past-year suicidal ideation among 1700 university students at Midwestern University in fall 2020, fall 2014, or fall 2013 found that rates of suicide ideation were not significantly higher in fall 2020 versus the earlier semesters. Nevertheless, rates of suicidal ideation in fall 2020 were significantly higher among sexual minorities other than heterosexual cisgender students. These data are consistent with Turner’s results [151] that identified sexual minorities as a high-risk group for mental health difficulties during the COVID-19 pandemic.

A longitudinal study conducted in Hong Kong [154] before, during, and after the first wave pandemic (baseline in September 2019 and follow-up until June 2020) to examine changes in suicidal ideation status among 1.491 adolescents, showed that the prevalence of suicidal ideation among young people was lower during and after the first wave than at baseline. The authors explained this result by referring to Hong Kong-specific socio-political dynamics prior to the outbreak of the pandemic. In fact, the pandemic may have reduced the socio-political tensions that afflicted young people by allowing them to devote themselves more to family and studies, thus reducing suicidal ideation 

As regards the psychiatric clinical population, data are too scarce to draw any conclusion. One study conducted in the northeastern United States evaluated the phenomenon of COVID-specific suicidal thoughts and behaviors among 143 adolescents with psychiatric disorders that were hospitalized [137]. Findings highlighted that COVID-specific suicidal ideation is common in high-risk youth and was associated with COVID-19-related negative emotions, elevated stress, and decreased public health guidance compliance. 

#### 4.3.2. Suicide Attempt

Three studies investigated the rate of anti-conservative attempts in the young population during the pandemic period.

One study was conducted in Japan [136] using monthly data on the total number of suicides obtained from suicide statistics compiled by the Ministry of Health, Labor, and Welfare. A total number of suicides per month (from March to May 2020) among children and adolescents under 20 years old was decreased. These findings are consistent with those reported in the study published by Odd and colleagues in England [148]. Authors compared the characteristics and rates of children dying by suicide between January 2020 and May 2020 with those in 2019. They used data from England’s National Child Mortality Database (NCMD) and they found no consistent evidence that child suicide deaths increased during the COVID-19 pandemic. 

Different findings were reported by Gracia and colleagues [143] extending the period under analysis. In their study, they analyzed data from the Catalonia Suicide Risk Code (CRSC), a secondary suicide prevention program that provides a well-established population-based registry of suicide attempts (SA). Comparing data of the first 12 months of Spain’s COVID-19 pandemic (March 2020 to March 2021) with data of the previous 12 months (March 2019 to March 2020), they observed an increase (25%) of suicide attempts among adolescents during the COVID-year. In addition, they noticed that the increase reached a rate of 195% in girls in the initial months of the school period (September 2020–March 2021). The discrepancy between these results may depend on differences in observation times, data collection methods, socioculturally mediated response to the pandemic, and the timing and intensity of the first pandemic wave in the different countries where the studies were conducted. 

#### 4.3.3. Emergency Department Attendance

In terms of emergency department attendance, in the United States, the proportion of mental health-related Emergency Department (ED) visits for children aged 5–11 and adolescents aged 12–17 years increased approximately by 24% among children and by 31% among adolescents in April 2020 [155].

In an observational retrospective study [150], Sokoloff and colleagues reviewed the number of patients younger than 18 years admitted to a New York City pediatric emergency department from March to May 2020 and during the same period in 2018 and 2019. Results showed that visits for suicidal ideation, suicide attempt, or self-harm increased by 100% although overall visits for psychiatric disorders decreased by 64%.

In Europe, during the first COVID-19 wave, two studies reported a decrease in psychiatric visits in emergency departments. In detail, a study conducted in France [147] reported a reduction of 50% in the incidence of admissions for suicide behaviors in pediatric and emergency pediatric units between January 2018 and June 2020. Similarly, in Italy, Davico and colleagues [141] examined all visits by patients under 18 years of age in the emergency department of two university hospitals (Turin and Rome) in the 7 weeks prior to 24 February 2020 and in the following 8 weeks of national lockdown. They reported a 72% decrease in the number of all visits with a 46.2% decrease in psychiatric visits without any significant changes in the prevalence of the primary reason for psychiatric visit (agitation, anxiety/mood disorders, and suicidality) or in the hospitalization rate. 

A general reduction of pediatric emergency departments’ admissions was also observed in Israel. Leff et al. [146] noticed a 60.84% decrease in the number of patients with mental health related diagnoses presenting to the pediatric emergency department during the pandemic (from March 2020 to May 2020) compared to the pre-pandemic period. These data suggested that young people with mental and behavioral health problems might be at risk for delayed detection of mental health disorders because, during the first wave of the pandemic, avoided presenting to emergency services when symptom onset occurs. 

In Turkey, Yalçın and collaborators [152] reported a change in the volume of pediatric emergency department visits and hospitalizations in the lockdown period (30 March to 31 May 2020) compared to the same non-lockdown period. They reported a reduction of 12% in emergency department visits and 41.6% in hospitalization, respectively. The rates of patients who suffered from mood disorders were found to significantly increase in the lockdown period than in non-lockdown. Although the rates of suicide attempts and aggressive behaviors were significantly lower in the lockdown group than in the non-lockdown group, younger patients were found to be at high risk for these conducts in the lockdown period.

The reduction of emergency department access was also reported by Ferrando [142] in a study that analyzed patients admitted for psychiatric evaluation between January and April 2020 in New York. Authors found a 43% decline in overall emergency psychiatric evaluation volume that, broken down by age group, corresponds to a reduction from 57% of children and adolescents in the pre-COVID-19 population to 32% during the COVID-19 period. These results are consistent with data provided by Pinho et al. [149] that showed a decrease of 52.2% in psychiatric emergency visits during the 45-days emergency state period (between 19 March and 2 May of 2020) compared with the same period of the previous year. When analyzing differences per age group, the younger age groups presented higher decreases of emergency visits when compared to the older age groups, with a maximum decrease of 59.2% for the 18–30 years group. 

Similarly, a French cross-sectional study [140] was aimed to assess temporal trends in suicide attempts among children and adolescents admitted to a pediatric emergency department in Paris, in the decade before and during the COVID-19 pandemic adjusting for annual and seasonal fluctuations. In this investigation, the number of suicide attempts among children decreased from 12.2 at the lowest level (July to August) in 2019, to 7.8 during the first lockdown period (March to April) in 2020 (—36%). On the other hand, during the second wave of the COVID-19 pandemic, the number of suicides substantially increased to 38.4 just before the second lockdown initiation (September to October) and to 40.5 (November to December) in 2020 (+116% and +229%). This aberrant dynamic of suicide attempts was independent of its annual seasonality and its trend over the 10 years. 

The same trend was reported in three studies performed, respectively, in the United States, Australia, and Canada. 

One study [153], examined trends in ED visits for suspected suicide attempts at three distinct phases of the COVID-19 pandemic, between January 2019 and May 2021, among subjects aged 12–25 years, included in the National Syndromic Surveillance Program (NSSP). Compared with the corresponding period in 2019, subjects aged 12–25 years had fewer ED visits for suspected suicide attempts during the period March–April 2020. However, by early May 2020, ED visit counts for suspected suicide attempts began increasing among adolescents aged 12–17 years, especially among girls.

A study conducted at the Royal Children’s Hospital in Melbourne [138] reported a 40% decrease in pediatric emergency department presentations (18,935 to 11,235) with a concurrent 47% increase in presentations to emergency department for mental health (809 to 1190) and a 59% increase for suicidality during the study periods between 2019 and 2020. Authors found that visits for suicidality were highest from June to September 2020 compared with 2019. Patients were aged between 7 and 18 years, but 86.2% of them were aged between 13 and 18 years. 

In addition, a retrospective analysis performed by Chadi et al. [139] in two large tertiary pediatric hospital centers in Canada between January 2018 and December 2020 showed an initial decrease in the number of mental health related ED visits at the beginning of the pandemic followed by a significant increase in the proportion of mental health related ED visits out of all adolescent ED visits starting in July 2020. Although they did not observe an increase in the overall number of mental health related ED visits, they found an increase in the proportion of mental health related ED visits.

In summary, most of the available evidence indicated that the frequency of suicide-related issues changed by the trend of the COVID-19 pandemic. Moreover, the worsening of the data in conjunction with the second wave suggests that the decline in rates of emergency department admissions and suicidal behaviors that occurred during the first lockdown may be ascribed to a failure in matching health needs with mental health services rather than to an actual decline in mental distress.

### 4.4. Role of Technology and Social Media 

During the COVID-19 pandemic internet use has globally increased as a consequence of self-isolation and social distancing. Internet networks and social media are the most used sources of information in the world: the easy access and the possibility of making real-time updates make them one of the easiest and most effective ways to provide and disseminate information. 

Social media platforms have also become helpful to maintain communication with friends and family members reducing isolation and boredom which have been associated with anxiety and long-term distress, therefore becoming important to reduce the psychological impact of the COVID-19 pandemic [156]. Moreover, web-based communication platforms have made it possible to carry out mental health-related therapeutic services in the form of telemedicine at a time when access to non-urgent care was blocked by the COVID-19 pandemic health emergency.

In this subsection, we will discuss scientific literature on the impact of web technologies on the mental health of young people focusing first on the use of the internet by the youth to address social distancing and the closure of schools and universities, and then on the role of telemedicine services for the mental health of the adolescents and young adults (Table 4).

#### 4.4.1. Impact of Web Technologies on the Mental Health of Young People during the COVID-19 Pandemic

More than 300 million students worldwide had to stop the regular attendance of school or university lessons because of the measure of contrast to the spread of the SARS-CoV-2. Nevertheless, information and communication technology allowed them to continue their education even when schools and universities were closed, all while maintaining social distancing [174]. 

It is important to note that, nowadays, the life of young people is closely related to social media, their “online environments” have become an integral part of critical youth developmental tasks [175]. These online environments reflect, reinforce, and complement psychological mechanisms, such as social comparison, self-disclosure, and impression management [176].

For these reasons, technology has played a crucial role during the quarantine for young subjects: they have increased the use of technological devices and social media platforms that have become fundamental for maintaining and enhancing socialization. 

However, we have also to consider the effects of such widespread use of technology in terms of disadvantages. In fact, the widespread of information implies the possibility that what is transmitted is not updated, is incorrect, or even false [177]. The spread of misleading information has become commonplace to the point of leading the World Health Organization (WHO) to warn of an ongoing “infodemic”: an overabundance of information, especially misinformation [178,179]. Within this context, social media could also exaggerate and boost alarmist information that can lead to psychological distress. Repeated exposure to media can cause depression, stress, anxiety, and fear in people with or without underlying psychiatric illness [180]. 

The excessive use of the internet itself can also generate a type of addiction: excessive or unlimited use can lead to “problematic internet use” or “pathological internet use,” which can result in marked distress and functional impairment in daily life, and comorbid psychiatric disorders, including substance abuse, attention deficit, and hyperactivity disorder, and depression [181,182]. Literature suggests that when exposed to stressful or traumatic events, young who have dysfunctional coping abilities are more prone to develop internet addiction [183,184,185]. 

Some studies analyzed the impact of web technology on mental health among young people during the COVID-19 pandemic, highlighting positive and negative effects as summarized in the following paragraphs. 

As suggested by the survey conducted by David and Roberts [160], the use of smartphones can attenuate the negative impact of social distancing on social connection and well-being. The authors tested 400 undergraduate students of a large U.S. university (52% female, mean age = 20) and they reported that social distancing is associated with lower social connection and subjective well-being and higher levels of stress and depression. They also found that smartphone use moderates the relationship between social distancing and social connection as a higher smartphone’s use improves an individual’s perceived social connection which is associated with better psychological well-being. This seems to be in line with a study performed in Italy to explore the relationships among anxiety, perceived vulnerability to disease, and smartphone use during the COVID-19 pandemic. For the 194 university students that were interviewed (mean age = 21.74), the use of smartphones seemed to mitigate the negative impact of social distancing on social connection and well-being. Unfortunately, a greater use of smartphones was also associated with a higher risk of addiction measured with the Smartphone Addiction Scale Short Version for Adolescents and Young Adults [161]. 

Another study aimed to verify the effect of social media on mental health during COVID-19 was performed in 248 international university students in The Netherlands [157]. Authors found that social media use had a positive impact on mental health outcomes during the COVID-19 pandemic in terms of improvement of depressive symptoms. 

These findings were confirmed by Sewall et al. [167] that conducted a four-wave panel study of U.S. young adults (N = 384; mean age = 24.5) to examine the association between mental health, objective digital technology use, and pandemic-related stressors. They reported that digital technology use did not contribute to increases in depression, anxiety, or suicidal ideation, in contrast with the popular notion that increases in technology use may be contributing to young people’s psychological distress during the pandemic. Rather, depression, anxiety, and suicidal ideation were mostly driven by pandemic-related impacts on well-being that had by far the largest effects on mental health.

Other studies reported contrasting findings. Wheaton [171] examined the relationship between media usage, susceptibility to emotion contagion, and emotional responses to the COVID-19 outbreak in 603 university students (mean age = 22.92 years). Results showed that consumption of media about COVID-19 significantly predicted the degree of COVID-19-related anxiety. 

Moreover, a large-scale Chinese cross-sectional study [158] conducted on a sample of 512 college students (mean age = 22.12 years) found that social media use was significantly associated with secondary trauma, depressive symptoms, and anxiety symptoms. Additionally, high social media use was positively associated with depressive symptoms when college students reported higher levels of COVID-19 stress. This finding suggests that COVID-19 stress moderates the relationship between social media use and depression. 

Similar conclusions were drawn by Li and colleagues [163] stating that social media were associated with negative mental health measures. Investigators analyzed data from 68,685 university students at two stages of the pandemic: soon after the start of the pandemic (T1) and 1 month later (T2). Comparing T1 and T2, social media use was significantly higher during the start of the pandemic. Heavy social media use (>3 h/day) at T1 was found to be a significant predictor of acute stress and anxiety symptoms, but not of depressive symptoms. The authors concluded that heavy social media may have a negative influence on short- and long-term mental health. 

Results obtained from an online survey and published by Shao and colleagues [168] went in the same direction. Authors evaluated individuals’ emotional state, regulation strategies, and outcome reappraisal and coping during the COVID-19 outbreak among 528 Chinese citizens (mean age = 35; SD = 10.65). They reported that hyperpersonal (social media-based) regulation strategies, such as disclosing and retweeting negative emotions, generate maladaptive effects. In particular, individuals who frequently disclosed pandemic-related feelings and retweeted negative emotions on social media reported less reappraisal of the stressful situation. In addition, stress and anxiety might cause a digital emotion contagion. 

In a population-based study performed in Hong Kong [173], a telephone survey was administered to 1070 adults (658 social media users and 412 non-users) between May and June 2020. Results showed that the relationship between social media usage and mental health could be moderated by age. In fact, younger participants obtained a large amount of information from social media that could easily trigger stress. Furthermore, time spent on social media was associated with depression symptoms during the COVID-19 pandemic maybe because spending more time on social media implies more exposure to COVID-19 news and a greater likelihood of experiencing the infodemic and emotional contagion.

These data are substantially consistent with those from a study conducted on 185 young adults, (mean age 21.59 years) from several countries [162] showing that an increase in young people of social media sites and streaming services use during the pandemic period resulted in compulsive internet use and gaming addiction that significantly predicted high scores of depression, loneliness, escapism, poor sleep quality, and anxiety related to the pandemic.

According to several investigations [186,187,188,189], increased use of internet gaming is linked with poor psychological adjustment. In fact, gaming has been used by adolescents and young adults as a coping mechanism to deal with the psychological distress of the pandemic. Gaming may be considered as an adaptive coping strategy in the short term, but it can become maladaptive if it evolves into the most used coping strategy for stress. 

#### 4.4.2. The Role of Telemedicine for the Mental Health of Young People during the COVID-19 Pandemic

In terms of health, social media platforms have also been an element of advantage during the COVID-19 pandemic because they permitted the possibility of arranging collaborative research projects, surveys, and multi-center studies and they also facilitated optimal service delivery while minimizing the hazard of direct person-to-person exposure with the use of telemedicine. Due to the pandemic, overburdened medical care frameworks have encountered a corresponding increase in demand for mental health services and countries have provided inadequate resources to meet this rush [190,191]. Since the early stages of the pandemic, social distancing has precipitated a radical transformation in the provision of all types of ambulatory healthcare from an in-person mode of delivery to mostly virtual telemedicine [192,193]. According to the World Health Organization, telemedicine is “the delivery of healthcare services, where distance is a critical factor, by all healthcare professionals, using information and communication technologies” [194]. When applied in the field of psychiatry, these methods are known as telepsychiatry.

Although the use of telemedicine was not uniformly adopted prior to COVID-19, with regard to mental health it is interesting to note that psychiatry was a telehealth pioneer: the earliest documented use of videoconferencing to support psychotherapeutic interventions and training occurred in the 1950s [195] and the term telepsychiatry was first proposed in 1973 [196]. With advances in technology, adoption of telepsychiatry slowly increased to become the second most practiced form of telemedicine in the world after teleradiology [197]. In 2015, the American Psychiatric Association formally convened a Committee on Telepsychiatry [198]. 

Given that young people are disadvantaged by mental illness and face further challenges related to the COVID-19 pandemic, it is crucial to deliver them appropriate mental health care. Furthermore, it has been suggested that digital technology may be particularly appealing to adolescents who are typically early adopters and regular users of new media [199]. For this reason, we decided to report the rapid implementation of telepsychiatry within adolescents and young adults in response to the COVID-19 pandemic.

Young people’s attitudes toward the use of technology have been investigated by Rauschenberg and colleagues [166]. They performed a cross-sectional panel study including a representative sample of individuals aged 16–25 years (N = 666; Mage = 21.3; assessment period: 5 May 2020 to 16 May 2020). There was some evidence that psychological distress, perceived social isolation, and lack of company, as well as COVID-19-related cognitive preoccupation, worries, and anxiety, were associated with the current use of mental health apps and individuals who experienced psychological distress were, across all levels of severity, more likely to report a positive attitude toward the use of mental health apps (more than those who did not report psychological distress).

Nicholas and collaborators [164] conducted an online survey to explore service provision, use, and quality following the adoption of telehealth among youth mental health service users (*n* = 308; aged 12–25) and clinicians (*n* = 92) of North-Western regions of Melbourne (Victoria) between 23 March and 11 June 2020. They highlighted that attendance rates were higher compared to the same period in 2019, even if service use was reduced. The majority of young people reported that telehealth positively impacted service quality and was significantly more likely to rate telehealth positively than clinicians. There was also high interest in continuing to use telehealth as part of care beyond the pandemic, supporting its permanent role in youth mental health care for a segment of service users.

Similar evidence has been reported by the qualitative study conducted by Palinkas and colleagues in the USA [165]: they interviewed 29 State Mental Health Authorities (SMHA) to determine the impact of the COVID-19 pandemic on the implementation of evidence-based policy and practice. The percentage of states reporting positive outcomes with telehealth implementation ranged from 80% to 100%, desire to continue the use of telehealth in the post-pandemic period ranged from 60% to 100%. For both parameters, the highest percentages were recorded in states with high rates of coronavirus positivity and high rates of unmet need. 

Also, a cross-sectional web-based survey conducted specifically among adolescents and young adults by Wood et al. [172] revealed that telehealth was highly acceptable among them and caregivers. A total of 55 patients and 123 caregivers rated telehealth as non-inferior to in-person visits with respect to privacy, communication, managing medication questions, discussing test results, mood, and mental health.

A few data are focused on patients with a diagnosis of eating disorders. Initial findings suggested that several services were able to adapt child and adolescent eating disorder treatment to virtual delivery [200,201].

Overall, the literature suggests that patients were disposed to undertake virtual therapy and recounted largely positive experiences with their eating disorder treatment. Authors found no differences in telehealth visit completion rates among patients [169,172] and some studies further suggested unexpected advantages of telehealth such as a greater level of familial involvement in treatment [159,202]. However, despite the advantages listed above, many patients reported preferring the face-to-face over the online type of appointment [159,169,170].

## 5. Conclusions

According to our results, the scientific studies on the impact of the COVID-19 pandemic on youth mental health are constantly growing in number, but, to date, the evidences obtained on this topic are still limited, fragmented, and partly controversial. At the level of scientific research, the pressure to publish data on the effects of the first pandemic wave and the consequent measures of social distancing may have produced a publication bias in favor of studies showing significant associations between the COVID-19 pandemic and negative outcomes of mental disorders of the youths. A further limitation of the value of these investigations is due to the defects in methods and data collection provoked by the urgency of submitting data to the scientific community. In fact, we found some common limitations in the methods of the studies considered in this review that can be summarized as follows: the selection bias linked to voluntary participation in studies performed through online surveys; the poor characterization of the samples included in the studies; the assessment of the outcomes, often self-measured with non-standardized tools and adopting different thresholds to verify the presence of clinically significant signs and symptoms; and the design of the studies, which is often retrospective or cross-sectional or, when longitudinal perspective, with a very short follow-up.

As expected, the methodological quality of the studies found is often mediocre, especially with regard to the selection of samples and the results to be evaluated and very short follow-ups. In addition, we have found very few targeted studies on new psychiatric diagnoses or the outcomes of clinical populations. Only a few large-scale epidemiological studies are highlighting the increase in cases of depressive and anxiety disorders in young people.

Based on the studies reported and discussed in this review, the following comments can be considered. As was to be expected, the COVID-19 pandemic has produced a significant impact on the mental health of young people. Like other traumatic events able to disrupt everyday life, it is linked to severe mental abnormalities in adolescents and young adults. Unlike other traumatic events, these negative effects derive both from the direct action on CNS functioning of neurobiological mechanisms elicited by SARS-CoV-2 infection, and from the indirect consequences of psychosocial processes induced by the measures enacted to prevent the spread of the virus and enhanced by the socio-economic fallout of the pandemic. The remarkable impact of the COVID-19 pandemic on the mental health of young subjects begins to emerge in several studies that have shown increases in the incidence of psychiatric symptoms and disorders. Symptoms and disorders with the most substantial increase in this population, especially in the female gender, are related to depression, anxiety, suicidal ideation, and eating disorders. In line with these data, access to the emergency room of adolescents and young adults with a mental health crisis has significantly increased in frequency. In this framework, technology appears to play two opposite roles: on the one hand, it is a tool for communication, sharing, education, and provision of care at distance, thus helping young people to preserve mental health during periods of isolation; on the other hand, technology is a potentially harmful factor that spreads fake or alarmist news and limits the interpersonal contacts in the real world.

To our knowledge, no other secondary literature study has provided a comprehensive review of scientific literature while taking into account all the aspects under consideration, therefore the main strength of this study is to have chosen to focus on a specific segment of the population but in relation to many aspects of mental health. Consequently, with regard to the objectives set, this work provides a useful and up-to-date summary, although still evolving, on a subject that concerns the clinical practice of different professional figures such as psychiatrists, psychologists, pediatricians, and general practitioners.

The main limitations of the study concern the inclusion criteria of the selected studies, chosen on the basis of the topic and not according to strict methodological criteria, the lack of extensive epidemiological data on the general population of young people and clinical subpopulations, and the choice of topics to be covered, on the one hand very broad and on the other focused only on some aspects of mental health of young people excluding others, such as resilience factors. This choice was motivated by the need to find a compromise between the topics most addressed in the literature, the available studies on this specific age group, and the length of the text in terms of readability. 

In our opinion, future research should promote studies on large samples of general and clinical populations of adolescents and young adults with a proper methodology, in order to provide a more reliable assessment and characterization of the damages produced by COVID-19 pandemic to the mental health of youths. Based on better knowledge of these effects and on evidences obtained in longer follow-up periods, it will be possible to provide to this vulnerable population effective and specific interventions aimed at minimizing the direct and indirect damage of SARS-CoV-2 infection.

In conclusion, the take-home message of the present study is to place attention in various clinical settings by attempting to detect the suffering of youth after the pandemic outbreak. This attention is required especially by professionals in contact with this stratum of the population, such as psychiatrists, psychologists, pediatricians, and general practitioners. The pandemic seems to have a significant impact, especially on the mental health of this specific segment of the population, especially in the female gender. Particular attention should be paid to depressive, anxiety, and eating symptoms, resources to be allocated to emergency rooms, and technological tools for telepsychiatry. 

## Figures and Tables

**Table 1:** Neuropsychiatric symptoms in the youth during and after the SARS-CoV-2 infection.

Authors	Study Design	States	Date of Data Collection	Sample Characteristics	Sampling Strategy/Data Collection Method	Outcomes
Cai et al., 2020 [78]	Longitudinal study	China	February–March 2020	N = 126 COVID-19 survivors Mage = 45.7	Online survey questionnaire	↔ No significant difference between older COVID-19 survivors and the younger survivors for depression symptoms. ↑ Younger participants have more emotional reactivity to infection, more anxiety symptoms, and more stress reaction symptoms than older survivors.
Blankenburg et al., 2021 [79]	Cross-sectional study	Germany	March–April 2021	N = 1560 students (Mage = 15 years) 1365 (88%) were seronegative, 188 (12%) were seropositive	Long-COVID19 survey questionnaire	↑ of neurocognitive, pain, and mood symptoms in the surveyed group of adolescents, most reported symptoms were insomnia, pain, fatigue, and concentration difficulties.
Blom-berg et al., 2021 [23]	Longitudinal study	Norway	February–April 2020	N = 312 COVID-19 survivors Mage = 46 years	Long term follow-up survey	52% of home-isolated young adults, aged 16–30 years, had symptoms: loss of taste and/or smell (28%), fatigue (21%), dyspnea (13%), impaired concentration (13%), and memory problems (11%).
Hampshire et al., 2021 [80]	Cross-sectional study	United Kingdom	January–December 2020	N = 81,337 participants (68,648 control)	Online questionnaire	Young adults as early as in their 20s showed large cognitive impairments in multiple domains particularly semantic problem solving, visual attention, and executive functions.
Roge et al., 2021 [81]	Longitudinal study	Latvia	1 July 2020, and 30 April 2021	N = 236 pediatric COVID-19 patients N = 142 comparison group patients	Clinical interview	2/3 of patients reported at least one persistent symptom 53% had two or more concurrent symptoms. The prevalence of persistent fatigue and cognitive symptoms significantly ↑ according to the study’s age groups: the highest rates were found among teenagers (14.7%, 1–4-year-olds versus 37.0%, 15–18-year-olds).

**Table 2:** Onset of new mental disorders and impact on pre-existing psychiatric disorders.

Authors	Study Design	States	Date of Data Collection	Sample Characteristics	Sampling Strategy/Data Collection Method	Outcomes
Al-Musharaf et al., 2020 [112]	Cross- sectional study	Saudi Arabia	18 May 2020 to 28 May 2020	N = 638 women, ages 18–39	Online survey	47.2% reported low emotional eating, 40.4% moderate, and 12.4% high. The main emotional eating indicators/predictors were fat intake (*p* = 0.004), number of meals (*p* < 0.001), sugar consumption (*p* < 0.001), body mass index (*p* < 0.001), stress (*p* = 0.004), energy intake (*p* = 0.04), and fast food intake frequency (*p* < 0.01). EE score correlated negatively with increased family income (*p* = 0.049).
Baiano et al., 2020 [108]	Longitudinal study	Italy	T0: 4 November 2019–17 February 2020 T1: 26 April–30 April 2020.	N = 25 University students Mage = 23.84	Online survey	↑ worriers at pre-lockdown and during lockdown conditions, ↑ anxiety sensitivity, and fear of mental health.
Cao et al., 2020 [101]	Cross- sectional study	China	-	N = 7143 Undergraduate age College students	-	About 24.9% of respondents experienced anxiety because of the COVID-19 outbreak. Protective factors: family income stability, living with parents, and social support. Negative factors: having relatives infected with COVID-19, economic stressors, academic delay, and effects on daily life.
Cellini et al., 2020 [120]	Longitudinal study	Italy	March 2020	N = 1310 University students and young workers aged 18–35 Mage = 23.91	Online survey	↓ sleep-wake rhythms markedly changed ↓ lower sleep quality. The decrease in sleep quality was stronger for people with a higher level of depression, anxiety, and stress symptomatology.
Dumas et al., 2020 [121]	Cross- sectional study	Canada	4–13 April 2020	N = 1054 14–18 years Mage = 16.68	Online survey	↑ in the frequency of both alcohol and cannabis use among adolescents.
Ellis et al., 2020 [92]	Longitudinal study	Canada	4–16 April 2020	N = 1054 Mage = 16.68	Online survey	↑ loneliness and depression, especially for adolescents who spend more time on social media.
Essadek et al., 2020 [96]	Cross- sectional study	France	27 April to 30 April 2020	N = 8004 University students Mage = 21.7	Online survey	43% of students suffered from depression (6.96% of severe level) 39.19% suffered from anxiety (20.7% of severe level) 42.94% from distress (16, 09% of severe level). Female scores were significantly higher than those of males.
Glowacz et al., 2020 [100]	Cross- sectional study	Belgium	17 April to 1 May 2020	N= 2871 adults 18–85 years (Mage = 33.67)	Online survey	↓ of living space, occupational activity, social contact, and alcohol use. ↑ anxiety, depression, and uncertainty than older participants.
Islam et al., 2020 [83]	Cross- sectionalstudy	Bangladesh	March 2020	N = 476 17 and older University students	Online survey	More than 2/3 of the students reported mild to severe depression (82.4%) and anxiety (87.7%). The prolonged unemployment, financial insecurity, and concern about academic performance were the most significant stressors.
Jiang et al., 2020 [105]	Cross- sectionalstudy	China	February 2020	N = 472 University students (17–22 years old)	Online survey	↑ levels in somatization, obsessive-compulsive disorder, interpersonal sensitivity, anxiety, phobic anxiety, paranoid ideation, and general severity index, during the pandemic.
Keel et al., 2020 [114]	Longitudinal study	USA	T1: 8–24 January 2020 T2: 15–24 April 2020	N = 90 university students Mage: 19.45 at T1; 19.71 at T2.	Online survey	↑ body weight, eating, screen time ↓ physical activity along with ↑ concerns about weight, shape, and eating since COVID-19. Longitudinal data indicated no significant change in weight, body mass index (BMI), or BMI category.
Khan et al., 2020 [84]	Cross- sectionalstudy	Bangladesh	April 2020	N = 505 19 or less (12.67%) 20–24 (78.42%) 25 or more (8.91%) College and university students	Online survey	28.5% of the respondents experienced stress, 33.3% anxiety, and 46.92% depression from mild to extremely severe. 69.31% had event-specific distress from mild to severe. Perceiving physical symptoms similar to the symptoms of COVID-19, fear of infection, financial uncertainty, inadequate food supply, no physical activity, and limited or no social activity had a significant association with stress, anxiety, depression, and post-traumatic symptoms. Excessive exposure to COVID-19 news in social and mass media had a significant association with depression, stress, psychological impact in terms of event-specific distress.
Liu et al., 2020 [97]	Cross- sectionalstudy	USA	13 April to 19 May 2020	N = 898 Aged 18–30 years	Online survey	↑ levels of depression (43.3%, PHQ-8 scores ≥ 10), anxiety scores (45.4%, GAD-7 scores ≥ 10), and PTSD symptoms (31.8%, PCL-C scores ≥ 45). ↑ loneliness, COVID-19-specific worry, and ↓ distress tolerance were significantly associated with clinical levels of depression, anxiety, and PTSD symptoms.
Ma et al., 2020 [93]	Cross- sectional study	China	3–10 February 2020	N = 746,217 College students	Online survey	The prevalence rates of acute stress, depressive and anxiety symptoms were 34.9%, 21.1% and 11.0%. COVID-19 epidemic factors that were associated with increased risk of mental health problems were having relatives or friends being infected. Students with exposure to media coverage of the COVID-19 ≥ 3 h/day were 2.13 times more likely than students with media exposure < 1 h/day to have acute stress symptoms.
Oosterhoff et al., 2020 [107]	Cross- sectional study	USA	28–29 March 2020	N = 683 adolescents Mage = 16.35	Online survey	98.1% reported engaging in at least a little social distancing due to social responsibility and not wanting others to get sick. Specific motivations for social distancing were differentially associated with adolescents’ anxiety symptoms, depressive symptoms, burdensomeness, and belongingness.
Parola et al., 2020 [82]	Longitudinal study	Italy	March–April 2020	N = 97 Young in a lockdown condition (aged 19–29).	Online survey	↑ Internalising and externalising symptoms while the lockdown measures were in place.
Puhl et al., 2020 [116]	Longitudinal study	USA	T1: 2018 T2: 2020	N = 584 participants T1 Mage = 21.9 years T2 Mage = 24.6 years	Online survey	Pre-pandemic experiences of weight stigma predicted higher levels of depressive symptoms (*p* < 0.001), stress (*p* = 0.001), eating as a coping strategy (*p* < 0.001), and an increased likelihood of binge eating (*p* < 0.001) among young adults during the COVID-19 pandemic but were unrelated to physical activity.
Qi et al., 2020 [90]	Cross- sectional study	China	8–15 March 2020	N = 7202 aged 14–18 years	Online survey	↑ prevalence of depression symptoms and anxiety symptoms. Only 24.6% of adolescents reported high levels of social support.
Seçer et al., 2020 [89]	Cross- sectional study	Turkey	Data collected during 15 days, no other information reported	N = 568 aged 14–18 years mean age = 16.4	Online survey	Fear of COVID-19 has a positive and significant effect on OCD. Considering possibilities, such as the speed of disease spread and the risk of death, it may be reasonable for adolescents to have washing and hoarding obsessions.
Scharmer et al., 2020 [117]	Cross- sectional study	USA	March–April 2020	N= 295 University students Mage= 19.7 years	Online survey	↑ ED pathology, but not compulsive exercise. Results from dependent samples *t*-tests indicated ↓ in exercise associated with COVID-19 (*p* < 0.001). Trait and COVID-19 intolerance of uncertainty moderated associations between COVID-19 anxiety and compulsive exercise and ED pathology. COVID-19 anxiety was more strongly related to compulsive exercise and ED pathology for individuals with lower intolerance of uncertainty.
Son et al., 2020 [97]	Cross- sectional study	USA	April–May 2020	N = 195 university students Mage = 20.7 years	Online survey	71% indicated ↑stress and anxiety 20% indicated it remained the same 9% mentioned ↓ stress and anxiety. Among those who perceived increased stress and anxiety, only 5% used mental health counselling services.
Tee et al., 2020 [91]	Cross- sectional study	Philippines	28 March–12 April 2020	N = 1879 Mage = 34.5 years	Online survey	16.3% of respondents rated the psychological impact of the outbreak as moderate-to-severe; 16.9% reported moderate-to-severe depressive symptoms; 28.8% had moderate-to-severe anxiety levels; and 13.4% had moderate-to-severe stress levels. Females had higher rates of psychological distress, anxiety, and depression due to the pandemic.
Wan Mohd Yunus et al., 2020 [85]	Cross- sectional study	Malaysia	April 2020	N = 1005 17 and older University students	Online survey	22%, 34.3%, and 37.3% of the university students scored moderate to extremely severe levels of stress, anxiety, and depression symptoms, respectively. The levels of stress, anxiety, and depression were significantly different according to age: younger students experienced more stress, anxiety, and depression symptoms compared with older ones.
Wang et al., 2020 [125]	Cross- sectional study	USA	May 2020	N = 2031 University students Mage = 22.88	Online survey	48.14% showed a moderate-to-severe level of depression, 38.48% showed a moderate- to-severe level of anxiety, and 18.04% had suicidal thoughts.
Yang et al., 2020 [108]	Cross- sectional study	China	28–30 January 2020	N = 8252 University students Mage = 17.9	Online survey	53.49% of the respondents were in a state of anxiety, while 46.83% were in a state of fear.
Zhou et al., 2020 [88]	Cross- sectional study	China	8–15 March 2020	N = 8072 students Mage= 16 range 12–18 years	Online survey	The prevalence of depressive symptoms, anxiety symptoms, and a combination of depressive and anxiety symptoms was 43.7%, 37.4%, and 31.3%, respectively
Baloch et al., 2021 [102]	Cross- sectional study	Pakistan	From 26 May to 6 June 2020	N = 494 College and university students below 18 years (9.1%) 19–25 (77.3%) above 26 (13.5%)	Online survey	41% of the respondents experienced minimal to moderate, marked to severe, and most extreme levels of anxiety. Female students were more anxious than male ones. The most prominent stressors are associated with online teaching, concerns about their academic performance, and completion of the current semester.
Bourion-Bédès et al., 2021 [103]	Cross- sectional study	France	May 2020	N = 3928 College and University students Mage = 21.74	Online survey	61% of students experienced anxiety during the lockdown. Female gender, having relatives infected with COVID-19, conflicts at home, difficulties isolating themselves, noisy environments, no direct outside access, delay in final examinations, reduced time for learning, and increased tobacco consumption were the main risk factors for anxiety.
Buckley et al., 2021 [113]	Cross- sectional convergent mixed methods (CMM) design	Australia	April until May 2020	N = 204 current (*n* = 93) and former (*n* = 111) athletes aged between 18 and 63 years Mage = 27.0	Online survey	Eating disorders were reported by 21.1% of participants. There was a significant difference between males and females (*p* = 0.018, r = 0.17). 34.8% (*n* = 69) self-reported worsened body image. 32.8% (*n* = 65) self-reported a worsened food relationship directly from COVID-19.
Czeisler et al., 2021 [122]	Cross- sectional study	Australia	15–24 September 2020	N = 1531 ≥18 years	Online survey	Younger adults reported ↑ adverse mental or behavioural health conditions than older adults.
Esposito et al., 2021 [124]	Longitudinal study	Italy	8 March–8 July 2020, versus the same period in 2019	N = 62 35 first episode of psychosis) FEP patients were hospitalized in 2020 27 in 2019	Clinical survey	↑ 29.6% in psychiatric hospitalizations for incident psychosis cases were observed. Patients with FEP in 2020 were significantly older than patients with FEP in 2021 and presented with significantly ↓ fewer substances abuse. Interestingly, patients presenting with FEP in 2020 were significantly older than patients with FEP in 2019.
Faize et al., 2021 [104]	Cross- sectional study	Pakistan	-	N = 342 University students Undergraduate age	Online survey	21.6% had mild, 9.4% had moderate and only 8.2% had severe anxiety. Students with severe anxiety reported psychological, social, and physical problems related to COVID-19, during the interview.
Kohls et al., 2021 [115]	Cross- sectional study	Germany	July– August 2020	N = 3382 University students Mage = 23.98	Online survey	33.0% reported binge eating at least once per week, 3.5% reported vomiting as compensatory behaviors minimum once per week, 0.1% usage of laxative minimum once per week, 25% diet or calorie food minimum once per week, 28.0% reported excessive exercising minimum once per week, 45.8% reported weight changes during the COVID-19 lockdown, 26.2% reported weight gain, and 19.6% reported weight loss. Of those reporting any weight change, over 63% attributed this to the pandemic and lockdown.
Luijten et al., 2021 [109]	Longitudinal Study	The Netherlands	April 2020 versus 2018	N = 2401 (2018) N = 844 (2020) 8–18 years	Online survey	During the lockdown, more patients reported severe Anxiety (RR = 1.95) and Sleep-Related Impairment (RR = 1.89) and fewer reported poor Global Health (RR = 0.36).
Mekonen et al., 2021 [86]	Cross- sectional study	Ethiopia	November 2020	N = 350 20 and older University students Mage = 24.70	Graduating class students available during the data collection period	The prevalence of stress, anxiety, and depression among graduating class students was 22.2%, 39.6%, and 40.2%, respectively.
Murata et al., 2021 [94]	Longitudinal study	USA	27 April to 13 July 2020	N = 4909 Mage = 40.3 Adolescents N = 583 Adults N = 4326	Online survey	Adolescents were significantly more likely to report moderate to severe symptoms of depression (55% versus 29%; *p* < 0.001), anxiety (48% versus 29%; *p* < 0.001), PTSD (45% versus 33%; *p* < 0.001), suicidal ideation or behavior (38% versus 16%; *p* < 0.001), and sleep problems (69% versus 57%; *p* < 0.001) compared to adults.
Padrón et al., 2021 [87]	Cross sectional study	Spain	-	N = 932 University students 18 and older	Online survey	Results indicated that students experienced considerable psychological problems during the lockdown, with ↑ of emotional difficulties in females and younger students than in male and older students, respectively.
Simone et al., 2021 [118]	Longitudinal study	USA	T1: 2010–1018 T2: April–May 2020	N = 720 Mage = 24.7	Online survey	Low-stress management, food insecurity, higher depressive symptoms, and financial difficulties were significantly associated with a ↑ of extreme unhealthy weight control behaviors (UWCBs). were significantly associated with a ↑ of UWCBs. Higher stress and depressive symptoms were significantly associated with ↑ of binge eating.
Sun et al., 2021 [98]	Cross- sectional study	China	March–April 2020.	N = 1912 University students Mage = 20.28	Online survey	67.05% reported traumatic stress, 46.55% had depressive symptoms, and 34.73% reported anxiety symptoms. Further, 19.56% endorsed suicidal ideation.
Trott et al., 2021 [119]	Longitudinal study	United Kingdom	T1: April–July 2019 T2: August–September 2020	N = 319 health club users Mage = 36.77	Online survey	↓ Exercise addiction scores (*p* = 0.034) ↑ Eating disorder symptomology scores (*p* = *<* 0.001) ↑ Leisure-time exercise (*p* = *<* 0.001) No differences in body dysmorphic disorder were found.
Wu et al., 2021 [123]	Longitudinal study	China	T0= 20 October 2019 T1 = 18 May 2020	N = 1825 adolescents	Clinical survey	↑ in adolescent Psychotic Like Experiences (PLEs) scores after the lockdown. We also found a positive correlation between changes in PLEs and changes in anxiety/depression. Furthermore, four PLEs trajectories were identified based on the report of PLEs at two time points: 60.4% with no PLEs, 9.3% remitted PLEs, 16.7% new PLEs, and 13.6% persistent PLEs.
Santomauro et al., 2021 [126]	Systematic review	-	1 January 2020 and 29 January 2021	N = 48 studies (46 studies met inclusion criteria for major depressive disorder and 27 for anxiety disorders) + additional 11 studies for major depressive disorder and seven studies for anxiety disorders	Systematic literature search	↑ in the COVID-19 impact, index were associated with an ↑ in the prevalence of major depressive disorder and anxiety disorders. For both disorders, females were affected more than males, and younger age groups were affected more than older age groups.
Taquet et al., 2021 [127]	Ecological study	UK USA	20 January 2020–19 January 2021 versus previous year	N = 5,186,451 patients Mage = 15.4 years	Electronic health records	↑ 15.3% incidence of eating disorders in 2020 compared with previous years. ↑ of relative risk from March 2020 onwards exceeding 1.5 by the end of the year. This increase occurred solely in females, especially in teenagers.

**Table 3:** Suicide-related issues in the youth during the COVID-19 pandemic.

Authors	Study Design	States	Date of Data Collection	Sample Characteristics	Sampling Strategy/Data Collection Method	Outcomes
Isumi et al., 2020 [136]	Ecological study	Japan	from March to May 2020	Population < 20 years old	Statistics compiled by the Ministry of Health, Labor and Welfare	No significant change in suicide rates during school closure compared to previous years (IRR = 1.15, 95% CI: 0.81–1.64).
Burke et al., 2021 [137]	Cross- sectional study	USA	13 March 2020, to 14 August 2020	N = 143 psychiatrically hospitalized adolescents Mage = 15.13	Participants were admitted to a psychiatric inpatient unit	COVID-specific suicidal ideation is common in high-risk youth and was associated with COVID-19-related negative emotions, elevated stress, and decreased public health guidance compliance.
Carison et al., 2021 [138]	Retrospective observational study	Australia	April– September 2020 versus the same period in 2019	Patients younger than 18 years	Electronic medical record review of all emergency department patients with mental health discharge codes	40% ↓ ED presentations 47% ↑ in MH presentations to ED during the study periods between 2019 and 2020. Suicidality presentation numbers were highest in June to September 2020 compared with 2019. Patients with a diagnosis of suicidality had a higher rate of re-presentation in 2020 compared to 2019.
Chadi et al., 2021 [139]	Retrospective observational study	Canada	January 2018 and December 2020	Adolescent 12–17 years old	Electronic health records With mental health discharge codes	↓ ED visits at the beginning of the pandemic followed by a significant ↑ in proportion of mental health related ED visits out of all adolescent ED visits.
Cousien et al., 2021 [140]	Cross- sectional study	France	2010–2021	Patients younger than 18 years	surveillance data	The number of suicide attempts among children ↓ from 12.2 at the lowest level (July to August) in 2019, to 7.8 during the first lockdown period (—36%). ↑ number of suicide attempts among children +116% before the second lockdown and +299%, early November to December 2020.
Czeisler et al., 2021 [122]	Cross- sectional study	USA	June 2020	N = 5470 participants aged 18–24 years	Web-based surveys	25% of the sample reported experiencing suicidal ideation related to the pandemic in the past 30 days, twice than in 2018.
Davico et al., 2021 [141]	Retrospective observational study	Italy	7 weeks prior to 24 February 2020 and in the following 8 weeks of national lockdown versus 2019	Patients < 18 years	Electronic medical record	72.0% ↓ of all pediatric ED visits (3395) compared with the corresponding period in 2019 (12,128), with a 46.2% decrease in psychiatric visits (50 versus 93).
Ferrando et al., 2021 [142]	Longitudinal study	USA	1 March–30 April 2020; N = 201) versus 1 January-28 February 2020 N = 355	Emergency Psychiatric Evaluation (EPE)	Electronic medical record	The most common psychiatric diagnoses and presenting symptoms during both periods were depression and suicidal ideation. Comparing the Pre-COVID-19 and COVID-19 periods ↓ in emergency psychiatric volume was observed in children and adolescents, but not adults.
Gracia et al., 2021 [143]	Ecological study	Spain	March 2020 to March 2021 versus March 2019 to March 2020	Adolescent 12–18 years old	Catalonia Suicide Risk Code (CRSC)	↑ 25% suicide attempts among adolescents during the COVID-year and they noticed that the increase in girls was prominent in the starting school period in the COVID-year, where it reached 195%.
Gratz et al., 2021 [144]	Cross- sectional study	USA	Fall 2020 Fall 2014 Fall 2013	N = 1700 university students	Screening questionnaires as part of the research requirement	Rates of suicide ideation were not significantly higher in Fall 2020 versus the earlier semesters.↑ Rates of suicidal ideation in Fall 2020 among sexual minority.
Hill et al., 2021 [145]	Cross- sectional study	USA	January–July 2020	N = 9092 participants Mage 14.72	Electronic health record of a large pediatric emergency department	↑ suicide-related matched times when COVID-related stressors were intensified.
Leff et al., 2021 [146]	Cross-sectional study	USA	10 March 2020, to 20 May 2020, versus the same period the year prior.	Patients younger than 18 years	Electronic medical record (EMR) system (EPIC™ 2010)	↓ of 60.84% of patients presenting mental health-related diagnoses, compared to the pre-pandemic period.
Mourouvaye et al., 2021 [147]	Retrospective observational study	France	1 January 2018 and 1 June 2020.	N = 234 Patients younger than 18 years	Patients were identified based on discharge codes	50% ↓ in the incidence of admissions for suicide behaviour.
Odd et al., 2021 [148]	Ecological study	England	January 2020–May 2020 versus January 2019–May 2019	Population under 18 years old	England’s National Child Mortality Database (NCMD)	No consistent evidence that child suicide deaths increased during the COVID-19 pandemic
Pinho et al., 2021 [149]	Retrospective observational study	Portugal	19 March and 2 May 2019 versus 2020	N = 2413 Adults admitted to the Psychiatric Unit of the emergency department	Administrative database that collects information of adult emergency department visits	52·2% relative ↓ on the total number of psychiatric emergency visits between the two periods with a maximum decrease of 59·2% for the 18–30 years group.
Sokoloff et al., 2021 [150]	Retrospective observational study	USA	7 March to 6 May 2020, and during the same period in 2018 and 2019.	PreCOVID *n* = 18,513; COVID *n* = 4068 Patients younger than 18 years	Data were collected Through routinely created hospital analytic reports	↑ 100% Visits for suicidal ideation, suicide attempt, or self-harms (*p* < 0.001) ↓ 64% visits for psychiatric disorders.
Turner et al., 2021 [151]	Cross- sectional study	Canada	June and July 2020	N = 809 participants aged 12–18 years Mage = 15.67	Social media advertisements	44% of adolescents experienced suicide ideation since the pandemic began, while 32% reported engaging in deliberate self-harm. SI and DSH were more common among youth who identified as transgender, gender fluid, or non-binary; who did not reside with both parents; and who reported frequent cannabis use or psychiatric concerns.
Yalçın et al., 2021 [152]	Longitudinal study	Turkey	30 March–31 May 2020, versus the same period in 2019.	Patients younger than 18 years	Electronic medical records	↓ 12% of PED visits and 41.6%, of hospitalizations in LP. ↑ anxiety and depressive disorders and bipolar disorders.
Yard et al., 2021 [153]	Retrospective observational study	USA	spring 2020; summer 2020; winter 2021 versus their corresponding reference periods in 2019.	Patients aged 12–25 years	Chief complaint terms and administrative discharge diagnosis codes.	↑ 50.6% suspected suicide attempt ED visits among girls aged 12–17 years than 2019; ↑ 3.7% suspected suicide attempt ED visits among boys aged 12–17 years. ↑ among adults aged 18–25 years throughout the pandemic compared with that during 2019
Zhu et al., 2021 [154]	Longitudinal study	Hong Kong	Baseline: 19 September; follow-up: 20 June	N = 1491 adolescents	A survey conducted in classrooms among volunteer participants	The prevalence of suicidal ideation was 24% and 21% among the participants before and during COVID-19, respectively. 65.0% remained non-suicidal, 14.0% recovered from being suicidal, 10.7% newly reported being suicidal, and 10.4% remained suicidal.

**Table 4:** Role of technology and social media on mental health of young people during the COVID-19 pandemic.

Authors	Study Design	States	Date of Data Collection	Sample Characteristics	Sampling Strategy/Data Collection Method	Outcomes
Misirlis et al., 2020 [157]	Cross-sectional study	The Nether-lands	6 April– 6 May 2020	N = 248 international university students	Online survey	Social media use has an inverse relationship with depressive symptoms and there was no statistically significant association between social media use and anxiety, loneliness, or the COVID-19 stressor (impact of events on student life).
Zhao et al., 2020 [158]	Cross-sectional study	China	24 March– 1 April 2020	N = 512 college students Mage = 22.12	Online survey	A higher level of social media use was associated with worse mental health. More exposure to disaster news via social media ↑ depression for participants with high (but not low) levels of the disaster stressor. Path analysis showed negative affect mediated the relationship of social media use and mental health.
Brothwood et al., 2021 [159]	Cross-sectional study	United Kingdom	March and November 2020	N = 14 young patients and N = 19 parents	Online survey	The experience of online Intensive Treatment Programme was rated positively. Parents rated it slightly higher (median = 8/10, IQR = 6.5–10) than young people (median = 6.5/10, IQR = 5–7).When online working was compared to face-to-face support responses were more varied. Young people generally found all online components of treatment less helpful than their parents.
David & Roberts, 2021 [160]	Cross-sectional study	USA		N = 400 university students Mage = 20 years	Online survey	Smartphones can mitigate the negative impact of social distancing on social connection and well-being
De Pasquale et al., 2021 [161]	Cross-sectional study	Italy	September 2020–January 2021	N = 194 University students Mage = 21.74	Online survey	Both men and women showed a high risk of smartphone addiction in SAS-SV. University students showed moderate trait and state anxiety in STAI and moderate perceived vulnerability to disease in PVD. The results showed that fear of COVID-19 and trait anxiety appear to be the predictors of STAI and PVD but not the predictors of risk of smartphone addiction (SAS-SV).
Fernandes et al., 2021 [162]	Cross-sectional study	India, Malaysia, Mexico, and the UK		N = 185 participants Mage = 21.59	Online survey	↑ use of social media sites and streaming services. Compulsive internet use, gaming addiction, and social media use significantly predicted high scores of depression, loneliness, escapism, poor sleep quality, and anxiety related to the pandemic.
Li et al., 2021 [163]	Longitudinal study	China	T1: 3 February–10 February 2020 T2: 24 March– 3 April 2020	N = 68,685 University students	Online survey	Heavy social media use (>3 h/day) at T1 was found to be a significant predictor of acute stress and anxiety symptoms, but not of depressive symptoms. Heavy social media may have a negative influence on short- and long-term mental health.
Nicholas et al., 2021 [164]	Cross-sectional study	Australia	March–June 2020	N = 308 participants (aged 12–25) + N = 92 clinicians	Online survey	Telehealth positively impacted service quality and was significantly more likely to rate telehealth positively than clinicians.
Palinkas et al., 2021 [165]	Quali-tative study	USA	November 2020–May 2021	29 State Mental Health Authorities (SMHA)	Online survey	Telehealth implementation ranged from 80% to 100%. Desire to continue the use of telehealth post-pandemic ranged from 60% to 100%. For both, the highest percentages were recorded in states with high rates of coronavirus positivity and high rates of unmet need.
Rauschenberg et al., 2021 [166]	Cross-sectional study	Germany	5 May– 16 May 2020	N = 666 participants aged 16–25 years Mage = 21.3	Online survey	8% of youth met the criteria for moderate or severe psychological distress. Social isolation worries and anxiety and objective risk indicators were associated with psychological distress. Psychological distress, worries, and anxiety were associated with a positive attitude toward using mental health interventions.
Sewall et al., 2021 [167]	Cross-sectional study	USA	August–November 2020	N = 384 young adults Mage = 24.5 ± 5.1	Online survey	None of the objectively-measured digital technology use variables were positively associated with depression, anxiety, or suicidal ideation at the within- or between-person levels. Pandemic-related impacts on mental health had by far the largest effects on depression, anxiety, and suicidal ideation.
Shao et al., 2021 [168]	Cross-sectional study	China	7 February–18 February2020	N = 528 Chinese citizens Mage = 35	Online survey	Hyper-personal (social media-based) regulation strategies, such as disclosing and retweeting negative emotions, generate maladaptive effects: individuals who frequently disclose pandemic-related feelings and retweet negative emotions on social media reported less reappraisal of the stressful situation.
Shaw et al., 2021 [169]	Cross-sectional study	United Kingdom	March–July 2020	N = 43 participants: 12 patients, 19 parents/carers, and 12 members of staff	Online survey	Patients, families, and staff all preferred face-to-face appointments over virtual options (e.g., telephone calls or video calls). There was no difference between the service satisfaction before and during COVID-19.
Stewart et al., 2021 [170]	Cross-sectional study	United Kingdom	18 May–25 July 2020	N = 53 young people with any eating disorder N = 75 parents N = 23 clinicians	Online survey	↑ Satisfaction with treatment, good engagement, and ability to manage technology. Young people who had transitioned care, rather than started care virtually in lockdown, rated therapy as less effective. However, individual accounts of experience were more varied.
Wheaton et al., 2021 [171]	Cross-sectional study	USA	5 April–13 May 2020	N = 603 university students Mage = 22.92	Online survey	Greater susceptibility to emotion contagion ↑ depression, anxiety, stress, and OCD symptoms. Consumption of media about COVID-19 predicted anxiety about COVID-19, though results were not moderated by emotion contagion. Emotion contagion did moderate the relationship between COVID-19-related media consumption and elevated OCD symptoms.
Wood et al., 2021 [172]	Cross-sectional study	USA	16 March and 15 April 2020	N = 55 patients (Mean Age = 18 years) + N = 123 caregivers (Mean age = 48 years)	Online survey	Telehealth as non-inferior to in-person visits with respect to communication, medication management, and mental health care for patients and caregivers. A higher proportion of patients compared to caregivers found telehealth inferior with respect to confidentiality (11/51, 22% versus 3/118, 2.5%, *p* < 0.001). One-quarter (14/55) of patients and 31.7% (39/123) of the caregivers reported technical difficulties.
Yang et al., 2021 [173]	Cross-sectional study	Hong Kong	May–June 2020	N = 1070 participants	Telephone survey	Negative direct effect of social media use on depressive symptoms among older people (*p* = 0.04) but not among younger people (*p* = 0.55). The indirect effect via PTSD symptoms was significantly positive among both younger people (*p* = 0.02) and older people (*p* = 0.01). The indirect effect via social loneliness was significant among older people (*p* = 0.04) but not among younger people (*p* = 0.31).
